# Reno-protective effects of oral alkalizing agents in chronic kidney disease with aciduria: protocol for a randomized cohort study

**DOI:** 10.1186/s12882-020-01807-8

**Published:** 2020-04-22

**Authors:** Michiaki Abe, Tetsuya Akaishi, Mutsumi Shoji, Takuhiro Yamaguchi, Takashi Miki, Fumitoshi Satoh, Shin Takayama, Satomi Yamasaki, Kazuhiko Kawaguchi, Hiroshi Sato, Tadashi Ishii, Sadayoshi Ito, CKOALA study group, Toshiki Nakai, Koichiro Nishioka, Satoshi Hashimoto, Hideyasu Kiyomoto, Keisuke Nakayama, Masataka Kudo, Ryo Morimoto, Takashi Nakamichi, Susumu Ogawa, Mariko Miyazaki, Kota Ishizawa, Takehiro Numata

**Affiliations:** 1 Department of Education and Support for Regional Medicine, Tohoku University Hospital, Sendai, Miyagi Japan; 2 Division of Nephrology, Endocrinology and Vascular Medicine, Tohoku University Graduate School of Medicine, Sendai, Miyagi Japan; 3 Clinical Research, Innovation and Education Center, Tohoku University Hospital, Sendai, Miyagi Japan; 4 Clinical Physiology Center, Tohoku University Hospital, Sendai, Miyagi Japan; 5 Medical Affairs Department, Nippon Chemiphar Co., Ltd., Tokyo, Japan

**Keywords:** Chronic kidney disease, Oral alkalizing agents, Bicarbonate, Citrate, Single-centered and randomized cohort study

## Abstract

**Background:**

Aciduria caused by urinary excretion of acidic metabolic wastes produced in daily life is known to be augmented in patients with chronic kidney disease (CKD). To evaluate the reno-protective effect of oral alkalizing agents for the improvement of metabolic acidosis and neutralization of intratubular pH in the patients with mild stages of CKD. Also, to identify reno-protective surrogate markers in the serum and urine that can closely associate the effect of urine alkalization.

**Methods:**

In this single-centered, open-labeled, randomized cohort study, patients with CKD stages G2, G3a and G3b, who visited and were treated at Tohoku University Hospital during the enrollment period were registered. We administered sodium bicarbonate or sodium-potassium citrate as the oral alkalinizing agents. A total of 150 patients with CKD will be randomly allocated into the following three groups: sodium bicarbonate, sodium-potassium citrate and standard therapy group without any alkalinizing agents. The data of performance status, venous blood test, spot urine test, venous blood-gas test, electrocardiogram, renal arterial ultrasonography and chest X-ray will be collected at 0, 6, 12 and 24 weeks (short-term study) from starting the interventions. These data will be also collected at 1 and 2 years (long-term study). The samples of plasma and serum and early-morning urine at every visit will be acquired for the analysis of renal function and surrogate uremic biomarkers.

The recruitment for this cohort study terminated in March, 2018, and the follow-up period for all the enrolled subjects will be terminated in December, 2020. The primary endpoint will be the development of originally-defined significant renal dysfunction or the occurrence of any cerebrovascular disease in the short-term study. The secondary endpoint will be the same endpoints as in the long-term study, or the patients with significant changes in the suggested the surrogate biomarkers.

**Discussion:**

The findings of this study will address the importance of taking oral alkalizing agents in the patients with early stages of CKD, furthermore they could address any new surrogate biomarkers that can be useful from early stage CKD.

**Trial registration:**

Registered Report Identifier: UMIN000010059 and jRCT021180043.

The trial registration number; 150.

Date of registration; 2013/02/26.

## Background

Chronic kidney disease (CKD) is a chronic progressive disease related to eating habits and lifestyle [1–3]. These days, the increment of end-stage kidney disease (ESKD) is one of the biggest problems in the management of CKD patients [4]. If the patients with CKD are not properly treated, the disease may lead to cardiovascular events as well as ESKD and renal death [5, 6]. The treatment to ameliorate tubulo-interstitial dysfunction in the progression of CKD is important for preventing the final common pathway leading to ESKD [7, 8], which is yet to be verified by clinical research.

Generally, renal dysfunction may lead to metabolic acidosis and hyperkalemia, both derived from the accumulation of acidic metabolic wastes produced in daily life [9–11]. The acidic metabolic compounds, called uremic toxins (UTs), are the substances that primarily cause the aciduria [12, 13]. The accumulation of UTs is more common in CKD patients than in healthy individuals because of the disturbed urinary excretion from the damaged kidneys [14]. Some types of UTs are known to stimulate the ROS production, which may lead to further renal tubule-interstitial dysfunction [15, 16]. Physiological acidification in renal tubules is not harmful itself; however, we previously reported that a stronger acid condition in renal tubules could result in an increased production of reactive oxidative stress (ROS), which could be aggravated by albuminuria [17]. In this previous study with a CKD animal model, an oral alkalinizing agent also improved aciduria by neutralizing renal intratubular pH and alleviating the latent metabolic acidosis.

In several past reports, treatments with some alkalinizing agents (sodium bicarbonate and sodium citrate) ameliorated renal damage in CKD patients with eGFR < 60 ml/min/1.73m^2^ [18–20]. In addition, intake of fruits and vegetables also ameliorated the renal dysfunction and high blood pressure [21]. Oral alkalinizing agents are also suggested to be useful for prolonging the initiation of hemodialysis by some unknown mechanisms, but this possible effect is still to be scientifically established.

In this protocol report, we planned a single-center randomly-allocated cohort trial, entitled as “Estimating the efficacy of the Oral ALkalinizing Agents in CKD; *i.e.* CKOALA study (UMIN000010059)”, to verify the reno-protective effects of the oral alkalinizing agents in patients with mild stages of CKD, and also to seek renal protective surrogate markers, affected by the reno-tubular alkalization.

## Methods

### Aims

To verify the renal protective effects of oral alkalinizing agents by neutralizing the aciduria in the patients with mild stages of CKD.

## Objectives

Our objectives are as follows:

1. To confirm the protective effects of oral alkalinizing agents on renal function in patients with mild and moderate-stages of CKD.
2. To compare the reno-protective effects of two types of oral alkalinizing agents, sodium/potassium citrate and sodium bicarbonate in CKD patients. Their effects will also be compared with that in the standard therapy group without oral alkalinizing agents.
3. To seek for the new surrogate markers in the serum and urine associated with early renal damage that can be cancelled by oral alkalinizing agents.

### Overview of study design

This study is a single-center, open-label, randomly-allocated cohort study, with CKD patients in stages G2, G3a and G3b. To reduce the risk of sampling error, the enrolled patients will be randomly assigned based on the stratified random sampling process by using the random sequence generated by a computer software [22, 23]. The used variables for the stratification will be age (i.e. ≥65, < 65 years old), sex, presence of diabetes mellitus, and eGFR (i.e. ≥46, < 46 ml/min/1.73m^2^). With these four variables, the enrolled patients will be divided into 16 subgroups, and then randomly allocated into the below-described three cohort groups from each of the subgroups.

**Fig. 1:**
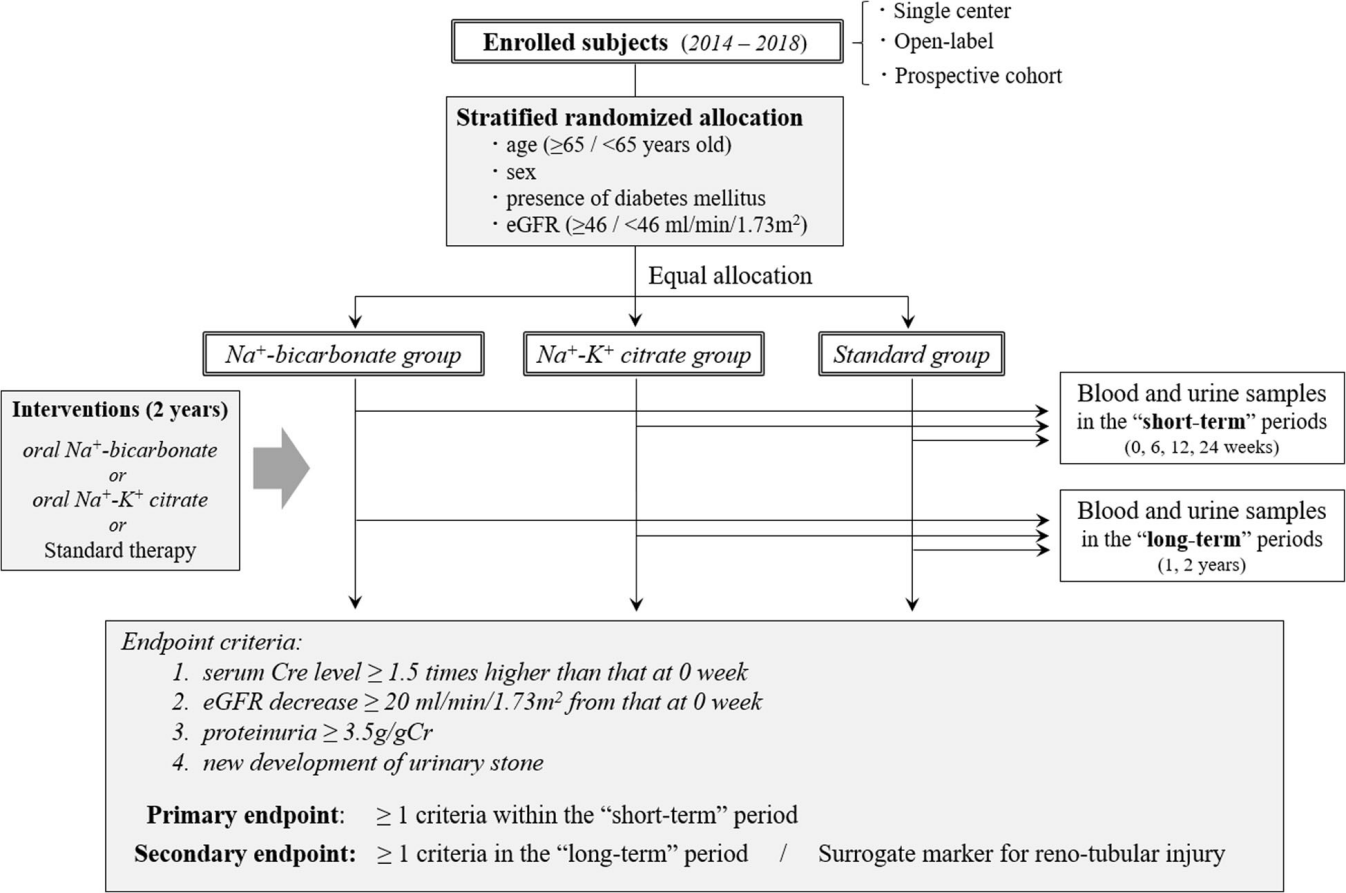
Overview of the CKOALA cohort study. Abbreviations: Cre, creatinine; eGFR, estimated glomerular filtration rate; gCr, gram adjusted by the urine creatinine level

The enrolled patients will be allocated into the following three cohort groups: 1) sodium bicarbonate group, 2) sodium-potassium citrate group, and 3) standard therapy group without any alkalinizing agents. Then, these three cohort groups will be measured for the targeted biomarkers in the serum and urine in the “short-term” study (i.e. at 0, 6, 12 and 24 weeks after starting the intervention) and in the “long-term” study (i.e. at 1 and 2 years after starting the interventions) to assess the effects of oral alkalinizing agents. As for the urine samples, both early-morning urine and spot urine will be collected. The above-described overview of this cohort study is shown in Fig. 1.

### Collected variables

At the time of registration, written informed consent is acquired from all the enrolled patients. At this time, the following information as to the patient’s background are also collected: birthdate, sex, past medical history, complications, medication history, allergic history, smoking history, amount of alcohol intake, body weight and height.

The routine physical examinations, medication usage, and occurrence of adverse events are confirmed at every check-up in both the short-term and the long-term assessments.

Other data concerning the quality of life (QOL), laboratory test, chest X-ray, and physiological studies are confirmed at the set periods shown in Table 1. In detail, cardiac-thoracic ratio and presence of congestion for chest XRs, severe arrhythmia, angina pectoris and acute myocardial infarction for ECG and intrarenal blood flow and renal size for renal US. The QOL was assessed by using the SF-8™ Health Survey (Standard, Japanese version) by QualityMetric Incorporation and Shunichi Fukuhara (iHope international) [24]. The details of each blood test category and urine test category are listed in Table 2.

**Table 1:** Items and timing of laboratory and physiological examinations for short-term and long-term assessments

	Short-term				Long-term	
	0 wk	6 wks	12 wks	24 wks	1 year	2 year
QOL (SF-8™)	○	–	–	○	–	–
Blood test						
Category I	○	○	○	○	○	○
Category II	○	–	–	○	△	△
Urine test						
Category I	○	○	○	○	○	○
Category II	○	○	○	○	△	△
ECG	○	–	–	○	○	○
CAVI, ABI	○	–	–	○	○	○
Renal US	○	–	–	○	○	○
Chest X-ray	○	–	–	○	○	○

Details of the categories I and II of both blood and urine tests are listed in the following Table 2. The circles show that the examinations will be performed at each time without exceptions. The triangles show that the examinations will be performed if the measured values in the short-term period showed abnormality and their follow-up is suggested to be desirable

**Table 2:** Details of categories I and II of both blood and urine tests

**Category I (blood test)**	
Complete blood cell count, hemogram, hemoglobin, hematocrit, reticulocyte, Cre, eGFR, uric acid, Na^+^, K^+^, Cl^−^, IgG, HCO3^−^, total cholesterol, LDL-cholesterol, HDL-cholesterol, triglyceride, glucose, HbA1c, CRP, BUN, Ca^2+^, phosphate ion, magnesium ion, Fe, TIBC, UIBC, total protein, albumin, AST, ALT, LDH, ALP, transferrin, α1-microglobulin	
**Category II (blood test)**	
Ferritin, whole PTH, plasma renin concentration, plasma aldosterone concentration, BNP, erythropoietin, bone specific alkaline phosphatase (BAP), insulin, cortisol, ACTH, leptin, adiponectin, endothelin-1 (ET-1)	
**Category I (urine test)**	
Creatinine, protein, albumin, pH, Na, K, Cl, urobilinogen, bilirubin, ketone body, occult blood, urinary sediments	
**Category II (urine test)**	
IgG, transferrin, retino-binding protein (RBP), α1-microglobulin, α2-microglobulin, NAG, Neutrophil Gelatinase-Associated Lipocalin (NGAL), KIM-1, L-FABP, 8-isoprastan, 8-OHdG, type IV collagen, ET-1, angiotensinogen, MCP-1, thioredoxin, IL-1β, IL-6, TNF-α, aldosterone, HCO3^−^, lactate, pH	

### Eligibility criteria for participants

The patients, 20 to 80 years of age with CKD stages G2, G3a and G3b who were treated at Tohoku University Hospital between March, 2014 and March, 2018, will be eligible for this study. As for the exclusion criteria, patients who were administered with any medications or drinks that could have possible alkalinizing effects, or patients with tolvaptan within 30 days prior to the registration will be excluded. In addition, patients with renal hypouricemia, hyperkalemia, diabetes insipidus, hypernatremia with unknown origins, morning urinary pH higher than 6.8, or serious complications of heart disease or liver disease will also be excluded. Patients with hyperuricemia, morning aciduria, or metabolic acidosis will not be excluded.

### Drug intervention

Administration of each oral alkalizing agent, sodium/potassium citrate or sodium bicarbonate, is started at 1.5 g per day. When early-morning urine pH is under pH 6.5, the drug will be increased to 3.0 g per day. When early-morning urine pH is over pH 7.2, the drug will be decreased or ceased up to less than pH 6.8.

### Censoring criteria

The censoring criteria as following; 1. hypernatremia, lower leg edema or early morning urine pH 7.2 or higher for 2 months even after taking a drug holiday, 2. Serum potassium level keeps over 5.5 mEq/L or under 3.5 mEq/L, 3. Adverse events such as exacerbation of symptoms on heart, liver or kidney, 4. subjects found to be ineligible after starting protocol treatment, 5. subject who want to stop or withdrawal the consent, 6. Subjects who do not visit because of moving etc. Data of censored subjects will be excluded.

### Endpoints

The primary endpoints will be **1)** the development of significant renal dysfunction, **2)** the occurrence of any kind of cerebrovascular disease between 0 and 24 weeks (short-term study) from starting the interventions. The significant renal dysfunction will be defined as the conditions that fulfill at least one of the following four criteria; 1. serum Cre level ≥ 1.5 times higher than that at 0 week, 2. eGFR decrease ≥20 ml/min/1.73m^2^ from that at 0 week, 3. proteinuria ≥3.5 g/gCr, 4. new development of urinary stones. The secondary endpoints will be the above-described criteria at 1 and 2 years (long-term study) from starting the interventions, and finding any kind of reno-protective marker associated with alkalinizing agents in both periods of study (Table 3).

**Table 3:** Primary and Secondary Endpoints

Endpoints	Period	
Primary	Short-term	1) The development of significant renal dysfunction 1. serum Cre level ≥ 1.5 times higher than that at 0 week 2. eGFR decrease ≥20 ml/min/1.73m^2^ from that at 0 week 3. proteinuria ≥3.5 g/gCr 4. new development of urinary stones 2) The occurrence of any kind of CVD and the death
Secondary	Lon-term	1) The development of significant renal dysfunction 2) The occurrence of any kind of CVD and the death
	Short-term & Lon-term	Reno-protective marker associated with alkalinizing agents in both periods of study, for example, category II (blood tests), categoryII (urine test), urine pH and unknown surrogate biomarkers of early renal damage, including uremic toxins and known metabolites measured by metabolomics.

### Sample size calculation

A sample size was decided based on a previous study evaluating the reno-protective effect of the sodium bicarbonate [20], in which the incidence of ESKD under treatment with and without bicarbonate were 6.5 and 33%, respectively. Based on this knowledge, we presumed the estimated incidence rate of ESKD in the sodium bicarbonate group and the sodium-potassium citrate group to be around 5 and 30%, respectively. To achieve the significant level (i.e. type-I error rate) of 5% (α = 0.05) and the statistical power of 80% (β = 0.2) with the equally allocated three groups, we estimated the ideal sample size in each group to be 50.

### Analysis

The measured variables in the three groups will be compared by the analysis of variance (ANOVA), followed by the Bonferroni’s post-hoc comparisons. If the variables show apparent non-normal distributions, Kruskal-Wallis test will be applied as a non-parametric test. Because of the simultaneous multiple comparisons, *p*-value < 0.01 will be regarded as statistically significant. The chronological change of each variable in each group will be also evaluated. Values in each pair will be compared by the paired Student’s t-test. If the variables show apparent non-normal distributions, Wilcoxon’s rank sum test will be adopted. Comparisons of outcome with dichotomous data (i.e. frequency) between the groups will be performed by either of chi-squared test or Fisher’s exact test, based on the achieved frequency in each cell. Lastly, to clarify the correlations between urine pH, both for the early-morning urine and spot urine, and serum creatinine levels, Spearman’s correlation coefficient for each pair will be calculated, followed by the test of no correlation.

## Discussion

Recruitment to this cohort study terminated in March, 2018, and the follow-up period for all the enrolled subjects will be terminated in December, 2020.

We will seek for new therapeutic strategies using oral alkalinizing agents for the early stage CKD patients in this CKOALA study. Based on the previous study that showed the reno-protective effects of the oral sodium bicarbonate in the animal CKD model [17], we hypothesized that administration of oral alkalinizing agents for the patients with mild and moderate stages of CKD would delay the development of ESKD. This cohort study will confirm the reported benefits of oral alkalinizing agents for suppressing CKD progression in actual human cases.

In addition to the expectation that this study will confirm the therapeutic effects of oral alkalinizing agents for CKD patients, it would also clarify the relationship between the urine pH and the chronic renal damage. Because the oral alkalinizing agents have been suggested to only alkalize the urine without changing serum pH, if the reno-protective effects of oral alkalinizing agents are confirmed, it will strongly suggest that aciduria is a definite risk factor to promote the renal damage in CKD patients.

One of the strengths of this study is that it will be conducted in a single center, which will enable us to exclude the risks of biases based on the differences between facilities and examiners. Meanwhile, there are some limitations. First, the number of enrolled subjects may remain low. If the results become negative due to inadequate sample size, we may extend the study period or plan additional randomized multi-center cohort study to achieve an adequate sample size. Another limitation is that this study will be an open-label trial. However, because the endpoints of this study are comprised of objectively measured serum and urine biomarkers that could not be affected by the preconceptions, biases based on the open-label style of this study to the given endpoints are not likely. Lastly, as for the patients’ compliance to the medications, we will interview the intake habit at all the occasions of their hospital visits.

## Data Availability

We informed the possibility that the samples and analysis results could be provided to other research institutes for secondary use, but personally identified information should not be used. The data that support the findings of this study are available from the Data center of Clinical Research, Innovation and Education Center (CRIETO) of Tohoku University Hospital, but restrictions apply to the availability of these data, which were used under license for the current study, and so are not publicly available. Data are however available from the authors upon reasonable request and with permission of the Data center of CRIETO.

## Abbreviations

CKD: Chronic kidney disease
ESRD: End-stage kidney disease
QOL: Quality of life
UT: Uremic toxins
ABI: Ankle brachial index
CAVI: Cardio ankle vascular index
ECG: Electrocardiogram
US: Ultrasonography
eGFR: Estimated glomerular filtration rate
LDL: Low-density lipoprotein
HDL: High-density lipoprotein
CRP: C-reactive protein
BUN: Blood urea nitrogen
TIBC: Total iron binding capacity
UIBC: Unsaturated iron binding capacity
AST: Aspartate transaminase
ALT: Alanine transaminase
LDH: Lactate dehydrogenase
ALP: Alkaline phosphatase
PTH: Parathyroid hormone
BNP: B-type natriuretic peptide
BAP: Bone specific alkaline phosphatase
ACTH: Adrenocorticotropic hormone
ET-1: Endothelin-1
RBP: Retino-binding protein
NGAL: Neutrophil gelatinase-associated lipocalin
KIM-1: Kidney injury molecule 1
L-FABP: Liver-type fatty acid-binding protein
MCP-1: Monocyte chemotactic protein-1
IL-1β: Interleukin-1β
IL-6: Interleukin-6
TNF-α: Tumor necrosis factor-α
CKOALA: Estimating the efficacy of the oral alkalinizing agents in CKD
